# An Assessment of Annual Mortality Attributable to Ambient PM_2.5_ in Bangkok, Thailand

**DOI:** 10.3390/ijerph17197298

**Published:** 2020-10-06

**Authors:** Nathaniel R. Fold, Mary R. Allison, Berkley C. Wood, Pham T. B. Thao, Sebastien Bonnet, Savitri Garivait, Richard Kamens, Sitthipong Pengjan

**Affiliations:** 1 Institute for the Environment, University of North Carolina at Chapel Hill, Chapel Hill, NC 27599, USA; nrfold@gmail.com (N.R.F.); mary.allison27@gmail.com (M.R.A.); berkleyw@live.unc.edu (B.C.W.); richkamens@gmail.com (R.K.); 2 The Joint Graduate School of Energy and Environment (JGSEE), King Mongkut’s University of Technology Thonburi, 126 Pracha Uthit Road, Bangmod, Thungkru, Bangkok 10140, Thailand; sebastien@jgsee.kmutt.ac.th (S.B.); savitri_g@jgsee.kmutt.ac.th (S.G.); sitthipong.ma@hotmail.com (S.P.)

**Keywords:** daily PM_2.5_/PM_10_ ratios, concentration-response coefficients, health burden, health benefit, Bangkok

## Abstract

Multiple studies indicate that PM_2.5_ is the most deleterious air pollutant for which there are ambient air quality standards. Daily concentrations of PM_2.5_ in Bangkok, Thailand, continuously exceed the World Health Organization (WHO) and the Thai National Ambient Air Quality Standards (NAAQSs). Bangkok has only recently begun to measure concentrations of PM_2.5._ To overcome this paucity of data, daily PM_2.5_/PM_10_ ratios were generated over the period 2012–2018 to interpolate missing values. Concentration-response coefficients (β values) for PM_2.5_ versus non-accidental, cardiopulmonary, and lung cancer mortalities were derived from the literature. Values were also estimated and were found to be comparable to those reported in the literature for a Chinese population, but considerably lower than those reported in the literature from the United States. These findings strongly suggest that specific regional β values should be used to accurately quantify the number of premature deaths attributable to PM_2.5_ in Asian populations. Health burden analysis using the Environmental Benefits Mapping and Analysis Program (BenMAP) showed that PM_2.5_ concentration in Bangkok contributes to 4240 non-accidental, 1317 cardiopulmonary, and 370 lung cancer mortalities annually. Further analysis showed that the attainment of PM_2.5_ levels to the NAAQSs and WHO guideline would reduce annual premature mortality in Bangkok by 33%and 75%, respectively.

## 1. Introduction

Globally, it is estimated that fine particles with aerodynamic diameters equal to or less than 2.5 µm (PM_2.5_) are responsible for approximately 3 to 9 million excess annual deaths [1,2,3,4,5,6,7]. It is thus not surprising that PM_2.5_ is considered one of the most dangerous pollutants [8]. Fine particles have the ability to enter the smallest airways and alveoli within the lungs, and ultrafine particles can subsequently diffuse into the bloodstream [9]. PM_2.5_ has been found to cause respiratory disease, specifically acute lower respiratory infection and chronic obstructive pulmonary disease, cardiovascular disease, specifically ischemic heart disease, cerebrovascular disease and stroke, and lung cancer [8,9,10,11,12].

Megacities around the world are rapidly expanding. This is particularly the case in Asian countries, where population growth is driving the need for continuous urbanization. Bangkok, the capital city of Thailand with a growing population of about 6 million inhabitants, [13] is on the cusp of emerging as the world’s next megacity. It is indeed witnessing major infrastructure development, which accounts for the majority of the country’s urbanization [14]. When one includes greater Bangkok, which extends beyond the formal Bangkok governmental boundary, there are more than 10 million people. Such expansion is associated with a number of environmental challenges, with air pollution becoming a notorious issue. In recent years, there have been increasing concerns over the situation of air quality in Bangkok. Pollution has risen to harmful levels resulting in unsafe concentrations of PM_2.5_, particularly during the dry season, as indicated by the Thai Pollution Control Department. Increased PM_2.5_ concentrations have been linked to consequential impacts that cause premature deaths [13,15]. Since 2012, pollution levels of PM_2.5_ have been monitored at various ambient air pollution stations around the country. However, the number of such stations is still limited compared to that of PM_10_. In 2015, only 12 stations were equipped to monitor PM_2.5_, 3 of which were located within the formal boundary of Bangkok. In 2018, the number of PM_2.5_ monitoring stations in Bangkok had increased to 19, thus providing better coverage of PM_2.5_ levels in the city. During 2016–2019, according to the 2018 Thailand State of Pollution Report, the 24-h NAAQSs standard of 50 µg/m^3^ was exceeded approximately 50 days per year. Maximum daily concentrations above 100 µg/m^3^ were observed during the dry season (November to April).

Health impacts related to unsafe air quality have been the subject of many studies, and focus on correlating particulate matter concentrations and premature mortality as well as related economic losses [8,12,14,15,16,17,18,19,20,21,22,23,24,25,26,27,28,29,30]. Some of these studies were performed using Thai populations [27,28,29,30]. Vichit-Vadakan et al. [27], in 2008, under the Public Health and Air Pollution program in Asia (PAPA), reported on the mortality impact of particle exposures with aerodynamic diameters equal to or less than 10 µm (PM_10_). They observed a 1.3% increase in mortality risk per 10 μg/m^3^ increase in PM_10_. This is higher than for similar exposures in some Western cities, as reported by Schwartz [23]. Wong et al. [29] investigated the excess risks associated with sulfur dioxide (SO_2_), ozone (O_3_), and PM_10_ also under the PAPA project for three main causes of mortality: non-accidental, cardiovascular disease, and respiratory disease. This study included Bangkok and three cities in China: Hong Kong, Shanghai, and Wuhan. The excess risks identified for Bangkok were found to be 2–5 times higher than those identified for China [29]. Another study by Guo et al. [30], in 2014, focused on assessing the excess risks associated with NO_X_, SO_2_, O_3_, and PM_10_ on mortality, including non-accidental, cardiovascular disease, and respiratory disease for 18 provinces in Thailand. They confirmed that air pollutants had significant short-term impacts on non-accidental mortality, and the effect was higher during the winter, compared to the rainy season. The study also highlighted that O_3_ is related to cardiovascular mortality, while PM_10_ is significantly related to respiratory mortality [30]. In the United States of America, Fann et al. [31] used the U.S. Environmental Protection Agency’s Environmental Benefits Mapping and Analysis Program (BenMAP) to investigate the health burden and benefits of PM_2.5_. However, to this day, the effects of PM_2.5_ on mortality in Thailand, specifically in Bangkok, have not been well-documented.

This study investigates annual mortality associated with PM_2.5_ in Bangkok based on available air quality monitoring data. There are currently no such studies with PM_2.5_. The specific objectives are (1) generate missing PM_2.5_ data by interpolation applied to existing PM_2.5_ and PM_10_ data to determine daily PM_2.5_/PM_10_ ratios, (2) investigate the association of PM_2.5_ with meteorological parameters, (3) identify relative risks and resulting concentration-response coefficients (β values) for all-cause, cardiopulmonary, and lung cancer mortalities, and (4) determine the annual mortality attributable to PM_2.5_ pollution utilizing BenMAP-CE.

## 2. Methodology

**Figure 1:**
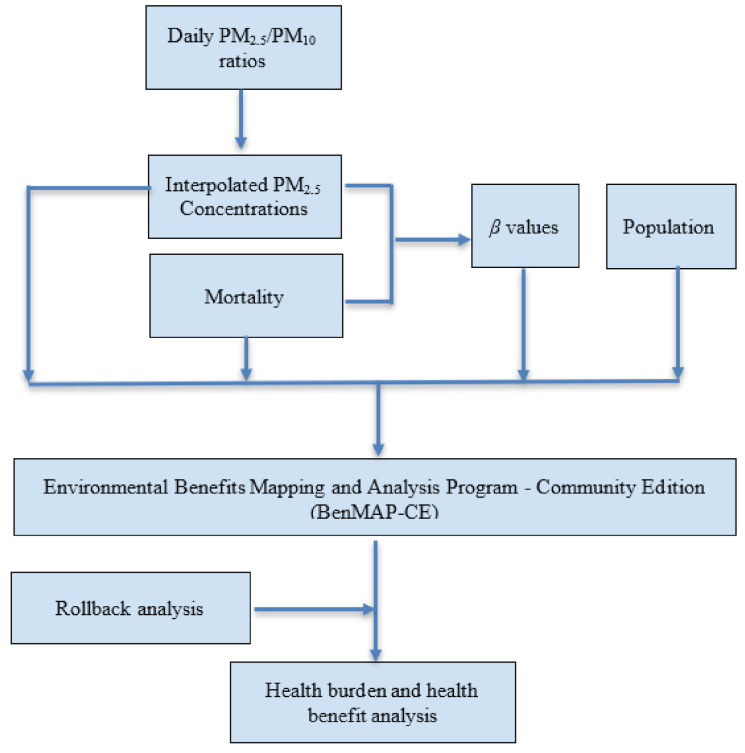
Workflow of the study procedure.

The specific steps to quantify mortality attributable to PM_2.5_ in Bangkok are illustrated in Figure 1. It begins with daily PM_2.5_/PM_10_ ratios, interpretation of PM_2.5_ values, and ends with a health benefits analysis.

### 2.1. Estimation of Daily PM_2.5_/PM_10_ Ratios and PM_2.5_ Interpolation

**Figure 2:**
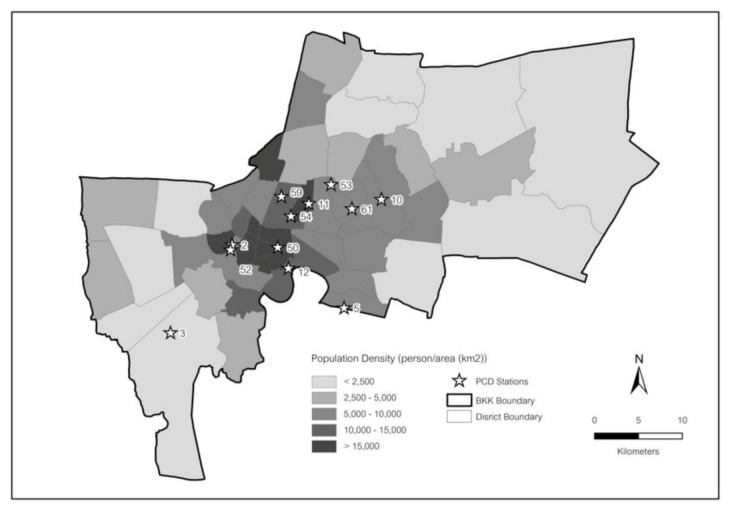
Population density and air quality monitoring stations over the study domain (stations include 59—PhayaThai; 02—ThonBuri; 03—Bangkhuntien; 05—Bang Na; 61—WangThonglang; 10—BangKapi; 11—DinDaeng; 12—Yannawa; 50—PathumWan; 52—ThonBuri; 53—LatPhrao; 54—DinDaeng).

As seen in Figure 2, fixed-site monitoring stations are clustered around Bangkok, especially in the central part of the city. Figure 2 additionally provides the associated sub-district population density nearby to each monitoring station. PM_2.5_ was measured by the Beta Ray attenuation method, following the United States Environmental Protection Agency (USEPA) reference method. In 2008, the USEPA designated this method as a federal equivalent method for measuring PM_2.5_ according to the US Federal register, 73 FR 22362, EQPM-0308-170 method. The equipment is from two main manufacturers, i.e., MetOne and Thermo. For the former, PM_2.5_ concentrations were recorded on an hourly basis. For the latter, PM_2.5_ concentrations were recorded every 10–15 min, and hourly average concentrations were calculated accordingly. Information on PM_2.5_ monitoring data and associated statistical values are presented in Table 1. We focused on data from 2012 to 2018 because all available monitoring stations collected data during these years.

During the years observed, the average PM_2.5_ concentration was 27.9 (±16.8) µg/m^3^ with maximum and minimum values of 170.7 µg/m^3^ and 2.15 µg/m^3^, respectively. The median concentration for PM_2.5_ was 24.0 µg/m^3^ with first and third quartiles of 16.9 µg/m^3^ and 35.0 µg/m^3^, respectively (interquartile range (IQR) = 18.0 µg/m^3^). As the quality of data varied between stations, the inclusion of specific data was based on the following criteria. A station’s PM_2.5_ data were accepted only if it contained at least 70% of the daily PM_2.5_ values in the original data. Daily means were only included if a station had at least 17 hourly PM_2.5_ measurements for each day. PM_2.5_ values were estimated from the average ratio of all available PM_10_ and PM_2.5_ values from other stations for that day. According to the USEPA, the PM_2.5_/PM_10_ ratio should fall between a range of 0.50–0.65 and must be applied to data from the same year [32]. Previous studies used a fixed annual PM_2.5_/PM_10_ ratio [33,34,35].

### 2.2. Estimation of Correlation between PM_2.5_ and Meteorological Conditions

Each ambient air quality station from the Pollution Control Department also measured a set of meteorological variables, including wind speed, wind direction, relative humidity, temperature, and rainfall, from 2012 to 2018. Wind direction and wind speed at each monitoring station were sampled 10 m above the ground with a 2-dimensional (2D) potentiometer wind vane and cup propeller for respective measurements. Temperature and relative humidity were measured 2 m above the ground using a multistage solid-state thermistor and a thin-film polymer capacitor for respective measurements. Rainfall was measured three meters above the ground with a tipping bucket. The equipment is from two main manufacturers, i.e., Met One and Thermo. Data were continuously recorded at an hourly frequency.

Meteorological variables had a seasonal influence on PM_2.5_ concentrations. Seasonality was defined for each month based on the weather conditions in a given month. Table 2 depicts the monthly averages of meteorological occurrences and associated PM_2.5_ concentrations. The dry/cool season was associated with lower temperatures and reduced cumulative rainfall (averages of 28 °C and 36 mm), as compared to the rest of the year. The hot season was associated with elevated temperatures (average of 30 °C) and was higher than the rest of the year. The rainy season was characterized by high levels of cumulative precipitation, averaging 225 mm during each month involved. June had characteristics of both the rainy and hot season, with an average temperature of 30 °C and rainfall of 197 mm. It was, therefore, assigned a mixed season classification of “Hot and Rainy”.

### 2.3. Mortality Data in Bangkok

Individual mortality records, including data on the location of death, age, sex, and primary causes of death from 2007–2016, were obtained from the Thailand Ministry of Public Health for the entire Bangkok metropolis area. There were approximately 460,000 non-accidental deaths during this time. The mean age was identified to be 64 (±20) years with a median age of 69 years, and first and third quartiles of 53 years and 79 years, respectively (IQR = 26 years). More men died during this period representing 59.3% of the total number of deaths. All deaths in the data set were classified as all-cause non-accidental, with cardiopulmonary disease and lung cancer contributing 15.7% and 3.2% of the mortalities, respectively. Mortality data in Bangkok are recorded by the Ministry of Public Health in the civil registration database. They are certified based on the death certificates. According to this information, it was assumed that the subjects registered in Bangkok had also lived and died in Bangkok. Each mortality datum was assigned a code classifying the cause of death according to the International Classification of Diseases, Tenth Revision (ICD-10) [36]. Previous epidemiological studies in China [10], India [8], and the United States of America [12,14,25,26] showed associations between PM_2.5_ concentration and cause-specific mortalities. Specifically, concordance was noted between PM_2.5_ pollution and cardiopulmonary diseases (ICD-10: I10-I15, I20-I52, I60-I70), and lung cancer (ICD-10: C33-C34, D022-D024). Here we also examined the mortality causes of cardiopulmonary disease and lung cancer, and these were coupled with all-cause (non-accidental) mortality (ICD: A00-R99) as a baseline reference. Mortality data were limited to an age range of 30–99 years since specific concentration-response variables from previous studies [10,25] focused on this age range. The age range 30–99 years accounts for 94.6% of the total number of deaths during 2007–2016, with approximately 440,000 deaths. It comprised 54.2% males and 45.8% females. For the age range 30–99 years, the mean age was 67 (± 15) years with a median of 70 years, and first and third quartiles of 56 years and 80 years, respectively (IQR = 24 years). Regarding cardiopulmonary mortality, ages in the range 30–99 years represented 96.3% of the total number of deaths in this category; the range 0–30 years accounted for the remaining 3.7%. For lung cancer mortality, the age range 30–99 years represented 98.4% of the total number of deaths in this category; the age range 0–30 years accounted for the remaining 1.6%. As a significant proportion of the mortalities observed in this study were attributable to the age range 30–99 years, the focus of the investigations of this study was on this age range. We determined a daily sum of each specific mortality category according to each ICD-10 mortality code, then determined each category’s incidence rate by dividing the number of deaths in each specific ICD-10 category by the total population during the same year.

### 2.4. Health Impact Assessment Using BenMAP

BenMAP-CE, a Geographic Information System (GIS)-based tool that estimates health impacts resulting from air pollution, was used to determine the links between PM_2.5_ concentrations and mortality. BenMAP-CE utilizes a health impact function that incorporates monitored air-quality data, population data, baseline incidence rates, and an effect estimate to calculate health impacts [15,37].

Relative risk *(RR)* is a ratio that compares, in this case, the mortality of a PM_2.5_ exposed group (at some PM_2.5_) to the mortality of a group with no PM_2.5_ exposure. The slope of the natural log of *RR* versus PM_2.5_ is called β, or the concentration-response (C-R) coefficient, and it is frequently used across different studies to compare the strength of the relative risk for a similar change in PM_2.5_ exposure (∆PM_2.5_). β can also be calculated from $\beta =\frac{ln\left(RR\right)}{∆PM2.5}$. A ∆PM_2.5_ of 10 μg/m^3^ is often used, but ∆PM_2.5_ can be used to estimate the reduction in mortality from an ambient value to some target or standard. In BenMAP-CE, β is used to calculate the change in the incidence rate, as a function of ∆PM_2.5_ as per Equation (1):

∆Y = Y_0_ (1 − e^−^^β^^∆PM^_2.5_) * *pop* (1)

where

ΔY is the change in incidence rate;
Y_0_ is the baseline incidence rate of the health effects;
β is the C-R coefficient;
*pop* is the exposed population, and
∆PM_2.5_ is the change in PM_2.5_ concentration to some target or health standard value.

The year 2016 was selected for the health burden and benefit analysis based on the completeness of data collected for that year. Baseline mortality data were obtained from the Ministry of Health and population data from the National Statistic Office. Concentration-response coefficients were derived as described above based on RR values retrieved from the literature. Estimates were also determined for Bangkok, as detailed in Section 3.3. The β values thereby obtained were used as input into Ben-MAP to assess the annual mortality endpoints considered in this study.

## 3. Results and Discussion

### 3.1. PM_2.5_ Interpolation

**Figure 3:**
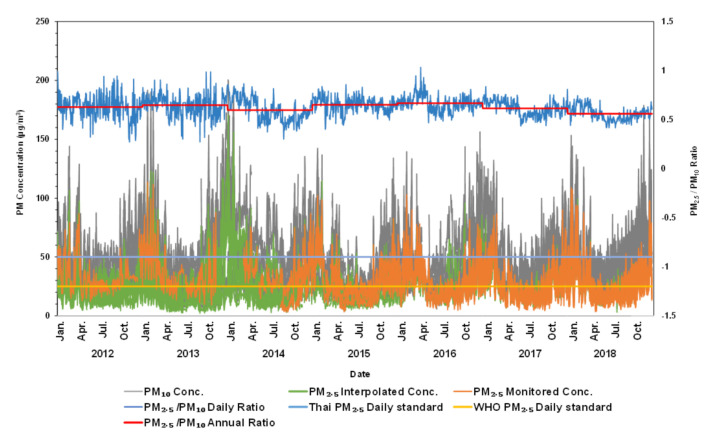
Daily average PM_2.5_ and PM_10_ concentrations, and PM_2.5_/PM_10_ ratios during 2012–2018.

Figure 3 shows the original available PM_2.5_ and PM_10_ data from 2012 to 2018, as well as annual ratios and daily ratios. The graph shows gap-filled data (PM_2.5_ interpolated in green) compared to non-gap filled data (PM_2.5_ original in orange). Further, the Thai and WHO PM_2.5_ air quality standards of 50 µg/m^3^ and 25 µg/m^3^, respectively, are denoted in Figure 3 to indicate days when air quality exceeded each set standard. PM_2.5_ data had a diminished density of data points during the first three years, which then increased through 2018. The above described daily ratio approach permitted the interpolation of more than 8000 PM_2.5_ values from all stations that were not previously available. The PM_2.5_/PM_10_ relationship was determined by generating a linear plot of PM_10_ as the independent variable and PM_2.5_ as the dependent variable, with PM_2.5_/PM_10_ as the slope of the line. Ten-fold cross-validation was run on the data utilizing a 90–10 model where 90% of the data were trained, and the residual 10% was tested using a generated PM_2.5_/PM_10_ ratio from the trained data. Cross-validation was carried out over 10 iterations, and the root-mean-squared error (RMSE) and coefficient of determination (R^2^) were averaged over the trials. When using the annual PM_2.5_/PM_10_ ratio to interpolate PM_2.5_ values, an accompanying R^2^ value of 0.634 (±0.042) and an RMSE of 15.137 (±2.51) µg/m^3^ were observed. In contrast, the daily ratio was proven to be significantly more accurate at predicting interpolated values with an averaged R^2^ value of 0.866 (± 0.018) and an RMSE of 3.607 (± 0.891) µg/m^3^. Data enhancement from the daily ratio allowed for more accurate predictions of PM_2.5_ concentrations, which further strengthened the relationships between variables analyzed in this study.

During the dry and cool months, PM_2.5_ concentration was high, and the annual ratio underestimated PM_2.5_ levels. However, during the rainy season, there were diminished concentrations of PM_2.5_, and the annual ratio overestimated PM_2.5_ values. Employing the daily ratio in place of the annual ratio permitted us to generate more accurate data, which improved mortality estimates using BenMAP-CE.

### 3.2. Correlation between PM_2.5_ and Meteorological Conditions

**Figure 4:**
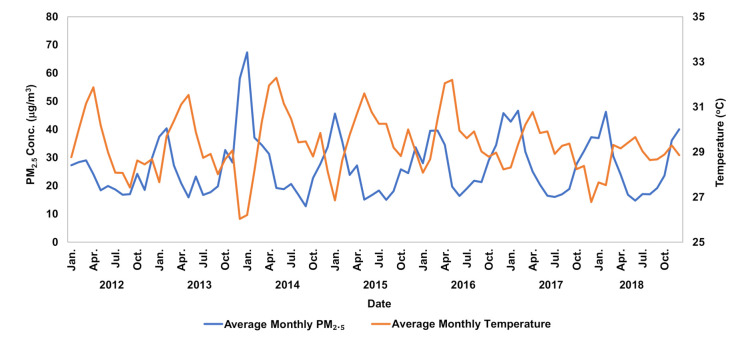
Monthly variation of PM_2.5_ concentrations and temperatures during 2012–2018.

**Figure 5:**
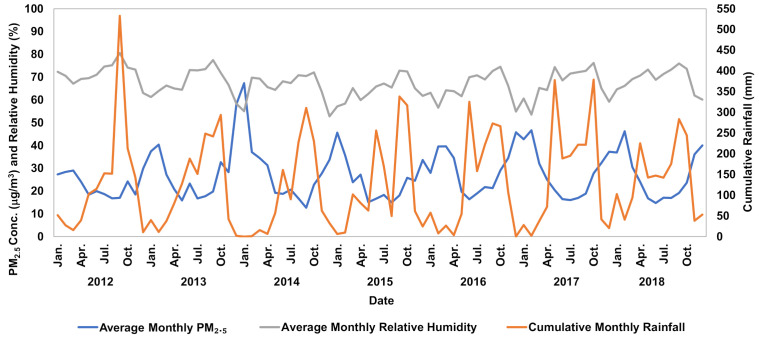
Monthly variation of PM_2.5_ concentrations and relative humidity with cumulative monthly rainfall during 2012–2018.

Observed correlations between particulate matter and meteorological factors showed a negative linear correlation between all meteorological variables and particulate matter. Relative humidity, temperature, and cumulative rainfall showed the strongest correlations with changes in pollutant concentrations, with Pearson correlation coefficients of −0.451, −0.240, and −0.201, respectively (Table 3). PM_2.5_ concentrations had an inverse relationship with changes in temperature and relative humidity (Figure 4 and Figure 5). Although the temperature in Bangkok varied less than 10 °C (mostly between 25 and 30°C) during the period observed, it was evident that as temperature increased, PM_2.5_ concentrations decreased and vice-versa. This trend was attributed to decreased mixing height from temperature inversions created by a change in temperature. These inversions trapped pollution and restricted vertical mixing, making pollution stagnant, thus increasing PM_2.5_ concentrations [38,39]. Increased rainfall reduced PM_2.5_ concentration through wet deposition by washing out the particles from the atmosphere [40]. Stagnation was perpetuated by lower wind speeds, although a decrease in wind speed did not cause an immediate increase in PM_2.5_ concentration because there was a brief lag period in which PM_2.5_ concentrations would build up. This lag became more significant throughout the study as stagnation in the atmosphere increased; overall, wind speed decreased by 31% from 2012 to 2018. Future climate is expected to become more stagnant, exacerbating air pollution and subsequent health problems [41,42]. The daily ratio accounted for the above meteorological occurrences and better depicted the daily fluctuations in PM_2.5_ concentration.

### 3.3. Health Benefit Analysis

In this study, we initially compared the US and Chinese values in BenMap, which represent different western and eastern global populations. These values were obtained by including other mortality risk factors such as sex, education, smoking, lifestyle, socioeconomic status, obesity, etc. As seen in Table 4, the Chinese β values [10] were much lower than the ones used for the United States [25]. The increment rollback function on Ben-MAP was applied to determine the impact of a 10 μg/m^3^ rollback in PM_2.5_ for the year 2016 (Table 4). This function reduced all PM_2.5_ observations by the same increment. A 10 µg/m^3^ rollback in Bangkok PM_2.5_ concentration utilizing the estimated Bangkok β values resulted in a 1.5%, 3.1%, and 4.1% decrease in annual mortality for non-accidental, cardiopulmonary disease, and lung cancer, respectively.

Differences in the number of avoided deaths in Bangkok were observed depending on the β values used. The number of avoided deaths in Bangkok, calculated from the Chinese β values [10], were found to be seven- (non-accidental), five- (cardiopulmonary), and four-times (lung cancer) lower when using β values reported in the literature for the United States [25].

**Figure 6:**
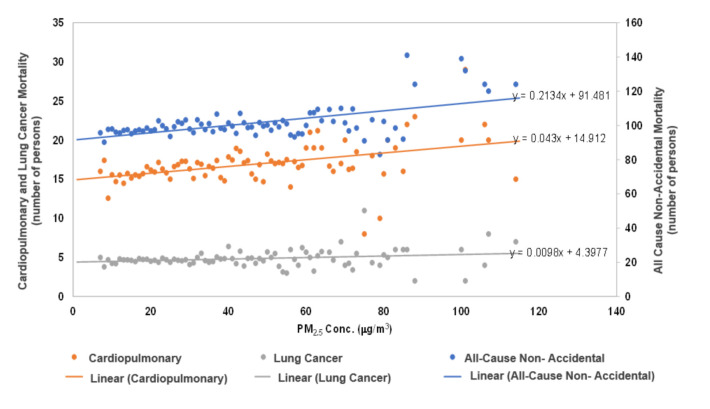
Relationship between All-Cause Non-Accidental, Cardiopulmonary, and Lung Cancer Mortalities and PM_2.5_ concentrations in Bangkok.

It is also possible to obtain simple RR values from the Bangkok population directly, by plotting Bangkok mortality data versus Bangkok PM_2.5_. Average daily mortality was computed for every 1 μg/m^3^ increase in PM_2.5_ over the range of concentrations observed. This produced trends, as shown in Figure 6. Specific relations were observed between PM_2.5_ concentrations and all non-accidental mortality, cardiopulmonary diseases, and lung cancer. The incorporation of interpolated PM_2.5_ concentrations allowed for improvement in the R^2^ significance for all the relationships between particulate matter and cause of death in this study, which provided strengthened conclusions. Initial R^2^ values between PM_2.5_ and all-cause non-accidental, cardiopulmonary, and lung cancer mortality classifications were 0.324, 0.184, and 0.098, respectively. Following the adjustment of PM_2.5_ data through interpolation, these values increased to 0.554, 0.364, and 0.162.

From the ratio of the mortality response on the y-axis and the y-intercept (unexposed PM_2.5_ mortality) in Figure 6, it was possible to compute *RR* values for a given ∆PM_2.5._ These values were then utilized to yield β values and estimates of mortality attributable to a given PM_2.5_ exposure. The authors are aware that although this approach is rudimentary and does not include other *RR* mortality factors, as per the Chinese and United States studies, it is very interesting that β values (standard deviations in parentheses) obtained in this manner (0.001743 (±0.0007458), 0.002284 (±0.003878), 0.003134 (±0.002754), for all-cause non-accidental, cardiopulmonary, and lung cancer mortality, were more similar with those determined from the Chinese population by Cao et al. [10]. Through generating our own β values, we were able to compare the associated population health risks from air pollution to other concentration-response values determined in studies from other countries.

These results suggest that populations in Bangkok and China tended to be more similarly affected by the same PM_2.5_ exposures, and were different from the US population. This observation is consistent with the study by Newell (2017) [43], which found regional differences in impacts on cardiorespiratory mortality and morbidity are observed for the same increase in particulate matter concentration. Populations in different regions of the world have a myriad of different traits (i.e., physiology, risk factors, lifestyle, etc.), which, apparently, influence their susceptibility and mortality response to PM_2.5_ exposures.

As reported in Table 5, anthropogenic PM_2.5_ levels above the background concentration of 2.15 µg/m^3^ in Bangkok using β values determined in this study, resulted in 4240 non-accidental mortalities, 1317 cardiopulmonary deaths, and 370 lung cancer mortalities. In comparison to meeting the Thailand annual standard of 25 µg/m^3^, meeting the WHO annual guideline of 10 µg/m^3^ would result in a significant reduction in premature mortality. While meeting the Thai annual standard is a goal that the Thai government is working towards, it is notable that this standard does not represent a PM_2.5_ level that is completely safe. Meeting the more stringent WHO annual standard is estimated to have a three-fold number of avoided non-accidental deaths. Meeting the Thai annual standard of PM_2.5_ would enable a 25% reduction in premature mortality, whereas meeting the WHO annual guideline would contribute a 71% reduction in premature mortality each year.

### 3.4. Uncertainty of the Analysis

BenMAP-CE required many data inputs. With each input, a layer of uncertainty is added that rests on the quality of the data. Mortality data had general limitations regarding the specificity of the district in which the mortality occurred. Because of this, the calculated incidence rates were generalized to the entire Bangkok province, as opposed to specific districts within the province. Of the available data, there were long periods in which PM_2.5_ values were not recorded. This was especially obvious between 2012 and 2014 when most monitoring stations only collected PM_10_ data. Missing PM_2.5_ concentration values were estimated using a daily PM_2.5_/PM_10_ ratio, which allowed for a continuous data set of PM_2.5_. For the BenMAP analysis, PM_2.5_ monitoring data from five monitoring stations in the year 2016 were used in accordance with the criteria of quality set out in this study. These five stations are located in central Bangkok and are not evenly dispersed. The Voronoi Neighbor Averaging (VNA) algorithm is an innate function of BenMAP-CE that was used to remediate the lack of PM_2.5_ monitoring stations in all 50 districts of the city. To determine the non-anthropogenic background concentrations of PM_2.5_, we used the lowest value recorded (2.15 µg/m^3^) from the 11 PM_2.5_ monitoring stations in Bangkok over 2012–2018. This procedure was followed because, to our knowledge, there is no established background concentration of PM_2.5_ in Bangkok. In regards to the health impact function, a key factor is the β value. Ideally, estimates of C-R coefficients should take into account other mortality covariates that could help provide additional insights into estimated Thai β values, such as male/female, BMI (body mass index), smoking/nonsmoking, alcohol intake, hypertension, educational level, individual socioeconomic status, other health conditions, medications, behaviors, etc. These well-known mortality determinants are missing in this analysis. Additional studies are needed to reduce their uncertainty.

## 4. Conclusions and Recommendations

This study used an innovative method for interpolating PM_2.5_ data based on seasonality and daily concentration changes in PM_2.5_ and PM_10_. Interpolating data points from this daily ratio, instead of annual ratios allowed for more accurate predictions of missing PM_2.5_ concentrations. With regard to human health, this study is the first health-related study linking annual mortality and PM_2.5_ in Bangkok. The results showed that by decreasing the annual PM_2.5_ concentration in Bangkok to the Thai NAAQS and WHO air quality standards, a consequential reduction of 1393 and 3159 in premature mortality attributable to unsafe PM_2.5_ levels can be achieved. Our results show that populations in Bangkok and China are more similarly affected by the same PM_2.5_ exposures than the population of the United States, and strongly suggest that regional β values be used in estimating PM_2.5_ mortality impacts.

Further studies should focus on investigating how PM_2.5_ may affect population health on episodic bases. In this study, meteorological information specific to Bangkok was gathered and investigated, and correlation analysis provided a rudimentary understanding of the potential of meteorological variables in assessing the concentration of PM_2.5_. This should be further investigated in future studies. Future epidemiological cohort studies should be carried out to determine concentration-response β values specific to Bangkok to more accurately quantify and model the relationship between PM_2.5_ levels and mortality. It is also very desirable to determine if these β values can be applied to non-Bangkok Thai populations.

## Figures and Tables

**Table 1:** Descriptive statistics of PM_2.5_ measurements in Bangkok during 2012–2018.

Year	No. of Station	Missing Data (%)	Statistical Values (µg/m^3^)	
			Mean (±SD)	Q1, Q3, and IQR
2012	1	90.3	33.7 (±14.0)	24.2, 38.5, and 14.3
2013	1	92.0	35.5 (±17.7)	22.5, 41.3, and 18.9
2014	2	88.5	30.6 (±16.1)	20.7, 39.4, and 18.8
2015	3	73.2	28.1 (±15.4)	17.5, 35.8, and 18.4
2016	5	61.4	27.7 (±14.8)	16.8, 34.3, and 17.5
2017	6	46.4	26.1 (±14.2)	15.6, 33.5, and 17.9
2018	10	22.5	27.2 (±15.3)	16.6, 33.7, and 17.0

SD: Standard deviation; Q1, Q3: First and third quartiles; IQR: Interquartile range.

**Table 2:** Seasonal variance in meteorological indicators and PM_2.5_ concentrations in Bangkok.

Season	Month	Average PM_2.5_ (µg/m^3^)	Average % Relative Humidity	Average Wind Direction (°)	Average Temperature (°C)	Average Wind Speed (m/s)	Monthly Cumulative Rainfall (mm)
Dry, Cool	January	40.8	62.0	165	27.7	1.1	40.9
Dry, Cool	February	39.1	62.7	169	28.9	1.1	14.3
Hot	March	31.0	66.7	185	30.2	1.2	47.0
Hot	April	26.7	65.5	186	31.1	1.1	72.6
Hot	May	17.9	67.2	194	31.0	1.1	132.2
Hot, Rainy	June	18.0	69.4	210	30.0	1.1	197.4
Rainy	July	18.1	70.8	219	29.3	1.1	151.3
Rainy	August	17.4	71.3	220	29.2	1.1	185.2
Rainy	September	18.2	74.7	212	28.7	0.9	314.6
Rainy	October	26.6	73.6	171	28.7	0.8	277.6
Dry, Cool	November	28.8	66.0	156	29.1	0.9	70.9
Dry, Cool	December	39.8	58.6	154	28.0	1.0	20.5

**Table 3:** Pearson correlations coefficient between particulate matter and meteorological factors.

Factors	PM_2.5_	PM_10_	Relative Humidity	Wind Direction	Temperature	Wind Speed	Rain
PM_2.5_	1.000						
PM_10_	0.944	1.000					
Relative Humidity	−0.451	−0.462	1.000				
Wind Direction	−0.353	−0.370	0.221	1.000			
Temperature	−0.240	−0.255	−0.170	0.291	1.000		
Wind Speed	−0.208	−0.295	−0.174	0.153	0.218	1.000	
Daily Rainfall	−0.201	−0.201	0.485	0.032	−0.261	−0.215	1.000

**Table 4:** Avoided deaths in Bangkok from a 10 µg/m^3^ rollback of PM_2.5_ in the year 2016.

	United States ^a^		China ^b^	
Health Endpoints	β Values (Standard Deviation)	Avoided Mortality	β Values (Standard Deviation)	Avoided Mortality
Mortality, All-cause non-accidental	0.00583 (±0.00096)	2772	0.000896 (±0.000538)	374
Mortality, cardiopulmonary	0.0122 (±0.00135)	1686	0.002547 (±0.006250)	316
Mortality, lung cancer	0.0131 (±0.00379)	291	0.00334 (±0.001758)	67

^a^: Pope et al. [25]; ^b^: Cao et al. [10].

**Table 5:** Health burden and avoided deaths in 2016 due to rollbacks to the Thai National Ambient Air Quality Standards (NAAQS) and the World Health Organization (WHO) guidelines.

Health Endpoint	Health Burden	Thailand Standard 25 µg/m^3^	WHO Guideline 10 µg/m^3^
	Deaths * (95% CI)	Avoided Deaths * (95% CI)	Avoided Deaths * (95% CI)
Mortality, non-accidental	4240 (1219–6938)	1393 (593–2691)	3159 (893–5248)
Mortality, cardiopulmonary	1317 (1065–1551)	360 (284–434)	959 (769–1140)
Mortality, lung cancer	370 (175–530)	102 (45–156)	270 (125–397)

* Specific for age 30–99.
